# Toxicity of clothianidin to common Eastern North American fireflies

**DOI:** 10.7717/peerj.12495

**Published:** 2021-11-19

**Authors:** Kirsten Ann Pearsons, Sarah E. Lower, John F. Tooker

**Affiliations:** 1 Department of Entomology, Pennsylvania State University, University Park, PA, United States of America; 2 Biology Department, Bucknell University, Lewisburg, PA, United States of America

**Keywords:** Neonicotinoid, Clothianidin, Flirefly, Lampyridae, Acute toxicity, Long-term toxicity, *Photuris versicolor*, *Photinus pyralis*

## Abstract

**Background:**

Previous research suggests that fireflies (Coleoptera: Lampyridae) are susceptible to commonly used insecticides. In the United States, there has been a rapid and widespread adoption of neonicotinoid insecticides, predominantly used as seed coatings on large-acreage crops like corn, soy, and cotton. Neonicotinoid insecticides are persistent in soil yet mobile in water, so they have potential to contaminate firefly habitats both in and adjacent to application sites. As a result, fireflies may be at high risk of exposure to neonicotinoids, possibly jeopardizing this already at-risk group of charismatic insects.

**Methods:**

To assess the sensitivity of fireflies to neonicotinoids, we exposed larvae of *Photuris versicolor* complex and *Photinus pyralis* to multiple levels of clothianidin-treated soil and monitored feeding behavior, protective soil chamber formation, intoxication, and mortality.

**Results:**

*Pt. versicolor* and *Pn. pyralis* larvae exhibited long-term intoxication and mortality at concentrations above 1,000 ng g^−1^ soil (1 ppm). Under sub-lethal clothianidin exposure, firefly larvae fed less and spent less time in protective soil chambers, two behavioral changes that could decrease larval survival in the wild.

**Discussion:**

Both firefly species demonstrated sub-lethal responses in the lab to clothianidin exposure at field-realistic concentrations, although *Pt. versicolor* and *Pn. pyralis* appeared to tolerate higher clothianidin exposure relative to other soil invertebrates and beetle species. While these two firefly species, which are relatively widespread in North America, appear somewhat tolerant of neonicotinoid exposure in a laboratory setting, further work is needed to extend this conclusion to wild populations, especially in rare or declining taxa.

## Introduction

In the United States alone, insects are estimated to provide over $50 billion in ecological services (Losey & Vaughan, 2006). Fireflies have great popular appeal and aesthetic and cultural value, but fireflies also contribute biological control of some pest species, including slugs and snails, which can be important agricultural pests (Godan, 1983; Lewis, 2016). Human activities, however, have put these services at risk by triggering global insect declines (Sánchez-Bayo & Wyckhuys, 2019). Some charismatic groups such as fireflies (Coleoptera: Lampyridae) may be at elevated risk of at least localized extinction due to ongoing human activities such as heavy pesticide use in and around their habitats (Reed et al., 2020).

Despite broad agreement that pesticides can pose a serious extinction threat to fireflies (Lewis et al., 2020), there is a very poor understanding of the direct toxicity of insecticides on fireflies. The most commonly applied classes of insecticides (neonicotinoids, pyrethroids, and organophosphates) are broadly neurotoxic to most insect taxa (Sparks, 2013), so fireflies are unlikely to be an exception. Indeed, full-strength organophosphate and neonicotinoid formulations are toxic to the aquatic firefly larvae *Luciola cruciata* and *Luciola lateralis*, respectively (Tabaru et al., 1970; Lee et al., 2008). Unfortunately, there have been no studies assessing how terrestrial firefly larvae respond to residual concentrations of these insecticides in soil, a likely route of exposure. Larvae of many common firefly species in North America are soil-dwellers that intimately interact with soil as they forage for prey and form protective molting chambers (Buschman, 1984; Lewis, 2016). These larvae inhabit forested, suburban, and agricultural soils, where neonicotinoid insecticides are often applied directly, or via coatings on crop seeds, to protect against pests (Knoepp et al., 2012; Douglas & Tooker, 2015; Simon-Delso et al., 2015). In these habitats, neonicotinoid concentrations in soil can range from less than 5 ng g^−1^ to over 4,000 ng g^−1^ (Knoepp et al., 2012; Schaafsma et al., 2015; Pearsons et al., 2021), concentrations that could plausibly influence behavior and survival of firefly larvae (Lee et al., 2008). Some indirect evidence suggests that firefly larvae are susceptible to neonicotinoids because adult lampyrid densities have been found to be lower where neonicotinoid-coated seeds were planted (Disque et al., 2019); however, to our knowledge, there have been no direct evaluations of how terrestrial firefly larvae respond to neonicotinoid-treated soil.

To assess the direct sensitivity of fireflies to neonicotinoid insecticides, we measured feeding behavior, development, and survival of larvae of two common North American firefly species—*Photuris versicolor* species complex and *Photinus pyralis* (Linnaeus 1767)—exposed to clothianidin-treated soil. We focused on clothianidin, one of the most widely-used seed- and soil-applied neonicotinoid and the primary metabolite of another commonly applied neonicotinoid, thiamethoxam (Douglas & Tooker, 2015). Generally applied to combat sucking and chewing insects, clothianidin disrupts insect central nervous systems, leading to paralysis and death (Simon-Delso et al., 2015). We exposed larvae to multiple field-realistic levels of clothianidin-treated soil for 30 to 100 days with the expectation that they would be sensitive to clothianidin at concentrations that have been detected in firefly habitats.

## Materials & Methods

### Chemicals

We acquired clothianidin from Chem Service (West Chester, PA, USA; purity ≥ 98%), and prepared stock solutions of 2  × 10^2^, 2  × 10^3^, 2  × 10^4^, and 2  × 10^5^ ng mL^−1^ clothianidin in acetone (Sigma Aldrich, St. Louis, MO, USA, ACS reagent, purity ≥ 99.5%). Pure acetone served as a control. We stored stock solutions at 4 °C and allowed them to reach room temperature (20 °C) before applying them to soils for the assays.

### Firefly collection and colony care

We ran toxicity assays on three separate cohorts of fireflies (Table 1): late-instar larvae from the *Photuris versicolor* species complex (*Pt. Photuris*), early-instar *Pt. versicolor*, and early-instar *Photinus pyralis (Pn. pyralis)*. Both *Pt. versicolor* and *Pn. pyralis* are relatively large-bodied (6–20 mm adult body length), widespread firefly species found throughout Eastern North America, and their populations do not appear to be declining (Lewis, 2016). Because both species spend 1–2 years in the soil as larvae and feed on soil invertebrates (*Pt. versicolor* are thought to feed on a diversity of soil invertebrates whereas *Pn. pyralis* larvae are considered specialists on earthworms; (McLean, Buck & Hanson, 1972; Buschman, 1984; Lewis, 2016)), they likely experience chronic contact and oral neonicotinoid exposure in contaminated habitats.

**Table 1:** Summary details of the firefly larvae (three cohorts of two species) used for toxicological assays.

Species	Instar group	Age (months)	Mean weight ± SD (mg)	# used in assay	Source
*Pt. versicolor*	late	>12	77 ± 17	30	wild-collected
	early	<3	9 ± 3	15	lab-reared
*Pn. pyralis*	early	<3	1.4 ± 0.5	75	lab-reared

Five of the late-instar *Pt. versicolor* were reared from eggs laid by a mated female collected in late July 2019 from the Bucknell University Chillisquaque Creek Natural Area (Montour Co, PA; 41°01′15″N, 76°44′53″W) , while the other 25 late-instar *Pt. versicolor* were wild-collected in summer of 2019 from multiple locations throughout Pennsylvania: Bald Eagle State Park (5 August; Centre Co, 41°00′44.0″N 77°12′54.3″W), Allegheny National Forest (24–25 June; Forest Co, 41°31′29.8″N 79°17′33.9″W), and Bucknell University Forrest D. Brown Conference Center (23–24 July; Union Co, PA; 40°57′28″N, 77°00′49″W). Specimens were collected in Bald Eagle State Park, Pennsylvania under permit SFRA-1907 to S. Lower and in Allegheny National Forest under firefly monitoring permit to the Pennsylvania Firefly Festival. Larvae were wild-collected at night by visually inspecting the ground for their faint glows. Larvae were identified to genus by external morphology (McLean, Buck & Hanson, 1972). After collection, we housed individual larvae in 16-oz clear plastic deli containers (11.5-cm diameter × 8-cm tall) lined with moist filter paper. Every 1–2 weeks, we provided each larva with one piece of cat food (Grain-Free Real Chicken Recipe Dry Cat Food, Whole Earth Farm™, Merrick Pet Care Inc., Amarillo, TX, USA), which had been softened in DI-water for 1 h (McLean, Buck & Hanson, 1972). After 24 h, we removed cat food and replaced the filter paper. Occasionally there was extensive fungal growth on the cat food, which could be fatal to *Pt. versicolor* larvae; in these instances, we gently wiped larvae with DI water and a delicate task wipe then transferred them to clean containers.

Early-instar *Pt. versicolor* and *Pn. pyralis* cohorts were reared from eggs laid in July 2020. On the evening of 10 July 2020, we collected 3 male and 2 female *Pt. versicolor* adults and 3 mated *Pt. versicolor* females. Flying *Pn. pyralis* males were collected and identified based on their characteristic “J” flash pattern (Lewis, 2016) while female *Pn. pyralis* were collected from nearby patches of short grass and were identified based on their flash pattern and similar morphology to the *Pn. pyralis* males (Lewis, 2016). Female *Pt. versicolor* were collected near *Pn. pyralis* females and identified based on their green-shifted flash color and morphology (Lewis, 2016). Additional *Pn. pyralis* males were collected to provision the mated *Pt. versicolor* females. We collected *Pt. versicolor* and *Pn. pyralis* in a residential area (State College, Centre Co, PA; 40°47′03″N, 77°52′25″W) into two separate 16-oz deli container “nurseries” kept at ambient room temperature (20–22 °C); each nursery contained a handful of moist sphagnum moss on top of moist soil (2-in deep; silt loam, collected from certified organic fields at the Russell E. Larson Agricultural Research Center at Rock Springs, PA, USA; 40°42′52″N, 77°56′46″W). Both *Pn. pyralis* females mated within a few minutes of collection.

Female *Pt. versicolor* and *Pn. pyralis* laid eggs within the following 3 days (50+ *Pt. versicolor* eggs and 100+ *Pn. pyralis* eggs; we did not attempt more accurate counts to avoid damaging eggs). Under ambient temperatures (20–22 °C), first-instar larvae of both species began to emerge three weeks after eggs were laid (5 August 2020). We kept *Pt. versicolor* larvae in the nursery chambers for two weeks, and then, after we observed significant cannibalism among larvae, moved them into individual soil-lined 1-oz polypropylene portion containers. As with larvae collected and reared from 2019, developing *Pt. versicolor* were fed moistened cat food (Grain-Free Real Chicken Recipe Dry Cat Food, Whole Earth Farm™, Merrick Pet Care Inc., Amarillo, TX, USA) in addition to pieces of freeze-killed *Lumbricus terrestris* (Josh’s Frogs, Owosso, MI, USA). As evidence of the hypothesis that *Pn. pyralis* larvae are specialist on earthworms, *Pn. pyralis* larvae did not feed on cat food but did feed gregariously on freeze-killed *L. terrestris*. Unlike *Pt. versicolor*, *Pn. pyralis* failed to thrive in isolation, so they were kept in the nursery chamber until starting the toxicity assay.

### Toxicity assay on Late-instar *Photuris versicolor*

We started the toxicity assay with late-instar *Photuris versicolor* on 22 June 2020. We used 1-oz polypropylene portion containers containing 8 g of dry soil (same soil source as nursery chambers) for our assay containers. To the soil in each assay container, we added 0.5 mL of the appropriate clothianidin stock solution, allowed the acetone to completely evaporate, then added two mL of DI water to moisten the soil and to achieve clothianidin concentrations of 0 ng g^−1^, 10^1^ ng g^−1^ soil, 10^2^ ng g^−1^ soil, 10^3^ ng g^−1^ soil , 10^4^ ng g^−1^ soil. We chose this concentration range (10^1^–10^4^ ng g^−1^ soil) to encompass the range of neonicotinoid concentrations in soil that have been measured in potential firefly habitats (Knoepp et al., 2012; Schaafsma et al., 2015; Pearsons et al., 2021).

**Figure 1:**
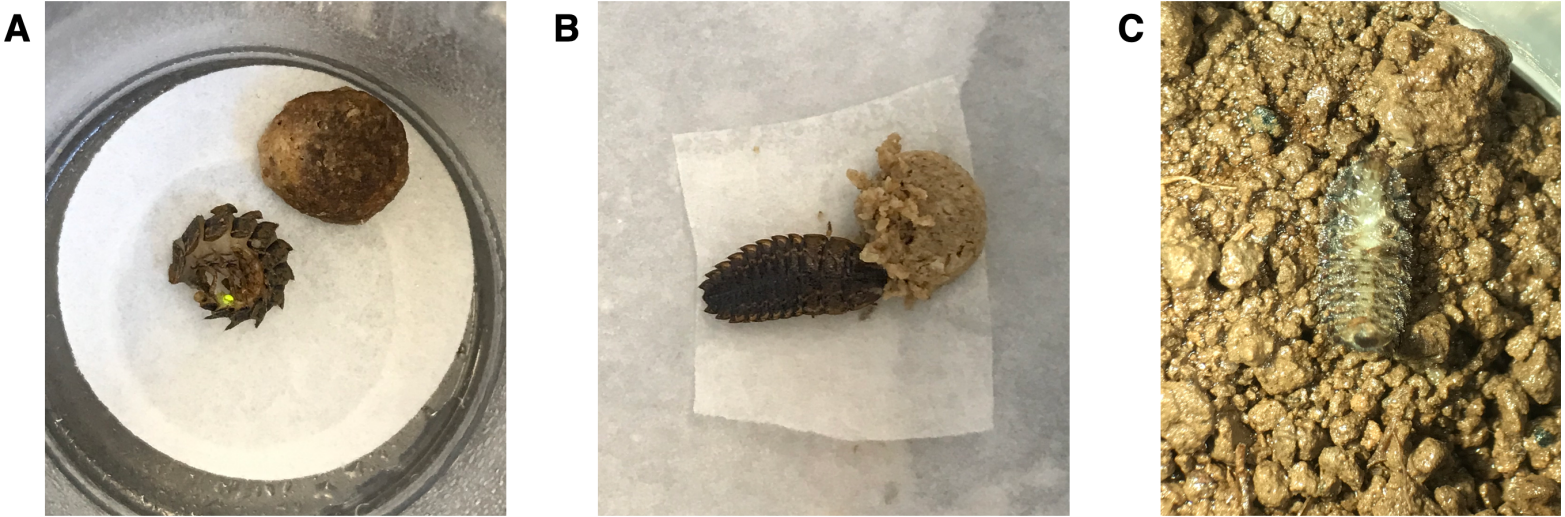
Healthy *Pt. versicolor.* larvae (A) demonstrating a typical “curl and glow” response after being prodded with blunt forceps and (B) feeding on moistened cat food. (C) An intoxicated *Pt. versicolor* larva on its back, unable to right itself.

After setting up assay containers, we weighed the late-instar *Pt. versicolor* and randomly assigned each to a particular clothianidin concentration (ensuring all larvae in each dose-set were sourced from the same location). All late-instar *Pt. versicolor* were over 12 months old at the start of the assay, and were over 10-mm long and >50 mg (Table 1). Each clothianidin concentration (0, 10^1^ ng g^−1^ soil, 10^2^ ng g^−1^ soil, 10^3^ ng g^−1^ soil , 10^4^ ng g^−1^ soil) was replicated six times (30 late-instar *Pt. versicolor* larvae in total). We recorded firefly status at 1, 4, and 24 h, and every day for an additional 99 d. Fireflies were categorized as dead (D), exhibiting a toxic response (T), or apparently healthy (A). A larva was assumed dead if it did not respond to gentle prodding with forceps. If a larva was flipped on its back and/or demonstrating repetitive twitching of its legs or head, it was recorded as exhibiting a toxic response (T). Fireflies were recorded as apparently healthy (A) if they exhibited a usual response to prodding from blunt forceps (Fig. 1A; quickly curled up on its side, often glowing). During the toxicity assay, we fed larvae once a week by carefully transferring individuals out of the assay containers into clean containers lined with moistened filter paper and containing a piece of moistened cat food. After 24 h, we returned fireflies to the assay containers and noted if the cat food had obvious signs of feeding (Fig. 1B). Feeding activity for each week was measured as a simple binary (0 = no obvious signs of feeding, 1 = obvious signs of feeding). At each status check, we noted if a firefly had constructed a protective soil chamber, then carefully dismantled the chamber to check larval status. Larvae often re-built soil chambers by the next day; if a larva built soil chambers on multiple consecutive days (feeding days as an exception), we noted this behavior as a “period of chamber formation.” Assay containers were kept in a dark drawer except when doing daily checks, and we misted containers with DI water as needed to keep the soil from drying out.

### Toxicity assay on early-instar *Photuris versicolor*

The toxicity assay with early-instar *Photuris versicolor* was similar to the assay with late-instar larvae, except we added half the amount of soil (4 g) and half the volume of clothianidin stock solutions (0.25 mL) to each assay container to achieve the same clothianidin concentrations (0, 10^1^ ng g^−1^ soil, 10^2^ ng g^−1^ soil, 10^3^ ng g^−1^ soil , 10^4^ ng g^−1^ soil). All early-instar *Pt. versicolor* were less than 3 months old and weighed between 3 and 15 mg. On 17 Sept 2020, we started trials with early-instar *Pt. versicolor* (three replicates at each concentration, 15 larvae in total), feeding them cat food once a week and recording their status at 1, 4, and 24 h, and every day for 10 d, then twice a week for an additional 90 d. Unlike for late-instar *Pt. versicolor*, we fed early-instars by directly placing moistened cat food in the assay containers (we removed the food 24 h later after noting if food had been damaged [1] or not [0]).

### Toxicity assay on early-instar *Photinus pyralis*

As with the early-instar *Pt. versicolor* assay, the *Photinus pyralis* assay was run in 1-oz polypropylene portion containers containing 4 g of soil with 0.25 mL doses of clothianidin stock solutions (to achieve 0, 10^1^ ng g^−1^ soil, 10^2^ ng g^−1^ soil, 10^3^ ng g^−1^ soil , 10^4^ ng g^−1^ soil). All early-instar *Pn. pyralis* were less than 3 months old and weighed between 0.6 and 2.4 mg. On 17 Sept 2020, we started the assay on early-instar *Pn. pyralis*, exposing larvae in sets of five (five larvae per container, three replicates at each concentration, 75 larvae in total), recorded their status at 1, 4, and 24 h, and every day for 10 d, then at least twice a week for an additional 20 d. We terminated the *Pn. pyralis* assay earlier than the *Pt. versicolor* assays due to an acarid mite infestation, which rapidly increased larval mortality across all doses. During the assay, we fed *Pn. pyralis* pieces of earthworm (*L. terrestris*) in the same manner that early-instar *Pt. versicolor* were fed cat food.

### Statistical analysis

We performed all statistical analyses in R (v4.0.4) (R Core Team, 2021). For each firefly cohort, we calculated median toxic concentrations (TC_50_) and median lethal concentrations (LC_50_) at 24 h, 7 d, and 30 d of exposure using probit analysis (LC_PROBIT from the “ecotox” package; Robertson et al., 2017; Hlina et al., 2019); for TC_50_ estimates, we included both sub-lethal and lethal responses, while LC_50_ estimates were based on mortality alone. To assess long-term survivorship across clothianidin levels, we used the Kaplan–Meier method (“survival” functions SURVDIFF and PAIRWISE_SURVDIFF; Therneau, 2021; Therneau & Grambsch, 2000). To determine how clothianidin exposure affected firefly behavior, we used non-parametric Mann–Whitney U tests (WILCOX.TEST) to compare feeding frequency and soil-chamber construction across clothianidin doses; we made pairwise comparisons using Wilcoxon rank sum tests with continuity corrections (PAIRWISE.WILCOX.TEST). As firefly larvae reduce feeding before pupation (McLean, Buck & Hanson, 1972), we excluded the two feeding events preceding pupation for feeding assessments.

## Results

### 24 h, 7 d, and 30 d TC_50_ and LC_50_ estimates

**Figure 2:**
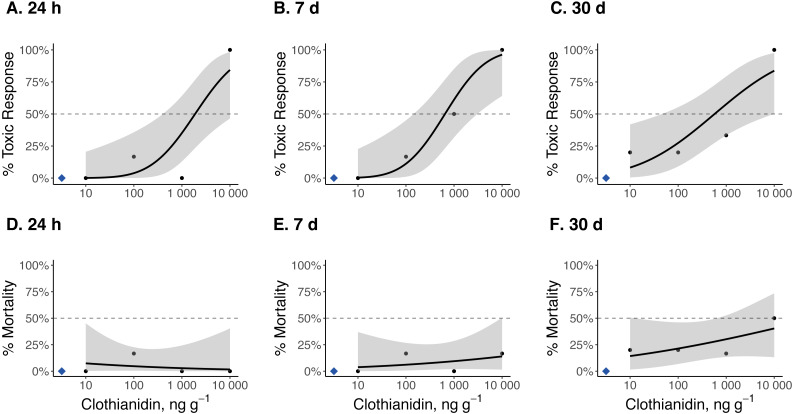
Dose–response curves for late-instar *Pt. versicolor* exposed to clothianidin-contaminated soil at 10, 100, 1,000, and 10,000 ng clothianidin per gram of soil (*n* = 6 larvae for each concentration). Toxic responses after (A) 24 h, (B) 7 d, and (C) 30 d, and lethal response after (D) 24 h, (E) 7 d, and (F) 30 d. Black dots in each panel represent mean responses at each insecticide concentration; the shaded area represents the 95% confidence interval for each curve. Blue diamonds represent the response of the control group. Dotted lines in each panel marks the 50% toxic response or mortality threshold.

**Figure 3:**
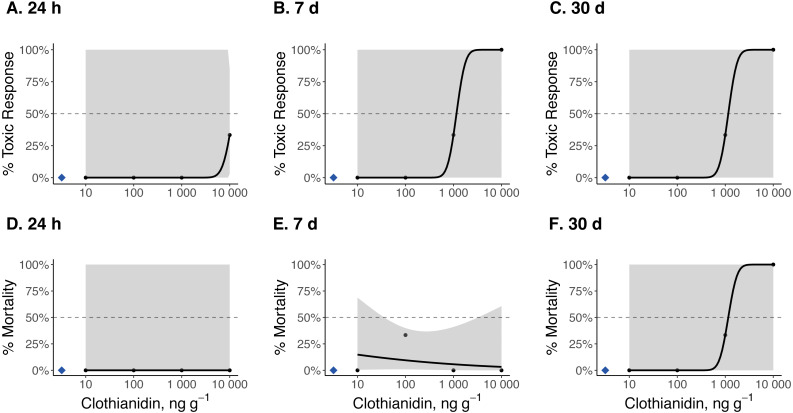
Dose–response curves for early-instar *Pt. versicolor* exposed to clothianidin-contaminated soil at 10, 100, 1,000, and 10,000 ng clothianidin per gram of soil (*n* = 3 larvae for each concentration). Toxic responses after (A) 24 h, (B) 7 d, and (C) 30 d, and lethal response after (D) 24 h, (E) 7 d, and (F) 30 d. Black dots in each panel represent mean responses at each insecticide concentration; the shaded area represents the 95% confidence interval for each curve. Blue diamonds represent the response of the control group. Dotted lines in each panel marks the 50% toxic response or mortality threshold.

**Figure 4:**
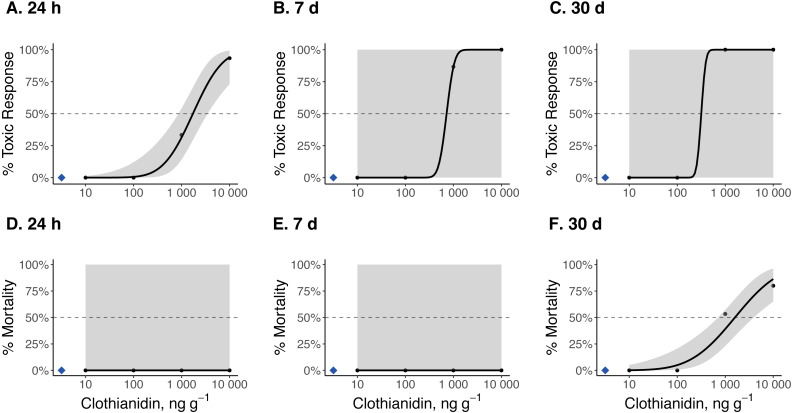
Dose–response curves for early-instar *Pn. pyralis* exposed to clothianidin-contaminated soil at 10, 100, 1,000, and 10,000 ng clothianidin per gram of soil (*n* = 3 sets of 5 larvae for each concentration). Toxic responses after (A) 24 h, (B) 7 d, and (C) 30 d, and lethal response after (D) 24 h, (E) 7 d, and (F) 30 d. Black dots in each panel represent mean responses at each insecticide concentration; the shaded area represents the 95% confidence interval for each curve. Blue diamonds represent the response of the control group. Dotted lines in each panel marks the 50% toxic response or mortality threshold.

Dose–response curves and estimated TC_50_ and LC_50_ indicate that *Photuris versicolor* and *Photinus pyralis* were surprisingly tolerant of exposure to clothianidin (Table 2 and Figs. 2–4). Reliable TC_50_ and LC_50_ estimates were limited by our small sample sizes and low acute mortality within the tested concentration range. Overall, TC_50_ values ranged from 500 ng g^−1^ to 2,000 ng g^−1^ while LC_50_ values exceeded our test limit (above 10,000 ng g^−1^).

**Table 2:** Estimated median toxic concentrations (TC_50_) and lethal concentrations (LC_50_) for *Pt. versicolor* and *Pn. pyralis* exposure to clothianidin-contaminated soil. 95% confidence intervals (CI) are based on probit analyses. CIs are not shown where data did not fit a cumulative standard normal distribution. n.r. = no response in tested range.

Species	timeframe		TC_50_ (ng g^−1^ soil)	95% CI		LC_50_ (ng g^−1^ soil)	95% CI
*Pt. versicolor,*	24 h		1882	136–10,000+		>10,000	–
late-instar,	7 d		648	144–3047		>10,000	–
*6 larvae / dose*	30 d		574	46–9895		>10,000	–
*Pt. versicolor,*	24 h		>10,000	–		n.r.	–
early-instar,	7 d		1169	–		>10,000	–
*3 larvae / dose*	30 d		1169	–		1169	–
*Pn. pyralis,*	24 h		1726	836–3486		n.r.	–
early-instar,	7 d		704	–		n.r.	–
*3 sets of 5 / dose*	30 d		316	–		1591	246–10,000+

### Firefly survival

**Figure 5:**
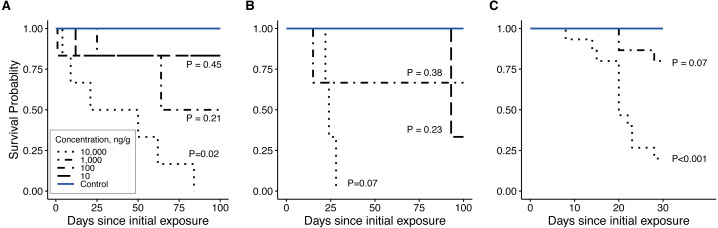
Survivorship curves. (A) late-instar *Pt. versicolor* (*n* = 6 per concentration), (B) early-instar *Pt. versicolor* (*n* = 3 per concentration), and (C) early-instar *Pn. pyralis* (*n* = 15 per concentration) at different clothianidin concentrations. *P*-values next to each line indicate the significance of reduced survivorship compared to the control (with a Benjamini–Hochberg correction for multiple comparisons). *P*-values were excluded where survivorship was 100% and perfectly overlapped with control values (100 ng g^−1^ in panel B, 10 and 100 ng g^−1^ in (C). Survival was significantly affected by clothianidin exposure (late-instar *Pt. versicolor*: ${\chi }_{4}^{2}=18$, *P* = 0.001; early-instar *Pt. versicolor*: ${\chi }_{4}^{2}=12.5$, *P* = 0.01; early-instar *Pn. pyralis*: ${\chi }_{4}^{2}=58.3$, *P* < 0.0001).

Clothianidin exposure significantly reduced long-term firefly survival at high concentrations (Fig. 5). Between one and four hours after initial exposure, half of the late-instar *Pt. versicolor* larvae and 87% of the early-instar *Pn. Pyralis* larvae exposed to the highest clothianidin concentration (10,000 ng g^−1^) began to exhibit toxic responses. By 24 h, all six late-instar *Pt. versicolor* exposed to the highest clothianidin concentration (10,000 ng g^−1^) exhibited a toxic response (Fig. 2A); these larvae never recovered and died by day 84. *Photuris* larvae were somewhat tolerant to lower clothianidin concentrations (10 ng g^−1^ or 100 ng g^−1^) and neither late- nor early-instar larvae exposed to low concentrations had significantly lower 100 d survival probability compared to controls (Figs. 5A–5B). All *Pt. versicolor* in the control treatment either pupated (2 out of 6 late-instar larvae) or survived through day 100 (4 out of 6 late-instar larvae, all three early-instar larvae). Although the experiment was terminated at 30 d due to the mite infestation, early-instar *Pn. Pyralis* exposed to 1,000 ng g^−1^ clothianidin showed marginally non-significant reduced survivorship (*P* = 0.07) while *Pn. pyralis* exposed to 10,000 ng g^−1^ clothianidin showed significantly reduced survivorship (*P* < 0.0001) compared to controls (Fig. 5C).

### Feeding behavior

**Figure 6:**
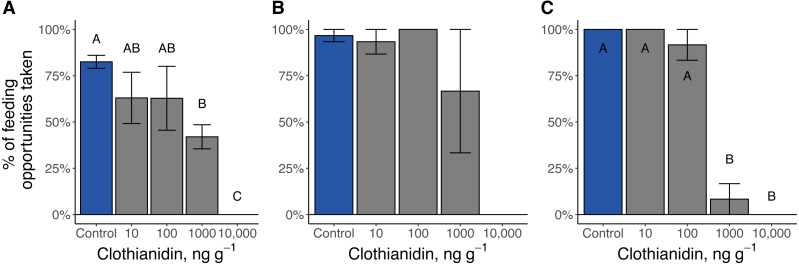
Percent of feeding opportunities taken by firefly larvae. (A) Late-instar *Pt. versicolor* larvae (${\chi }_{4}^{2}=15.8$, *P* = 0.003), (B) early-instar *Pt. versicolor* larvae (${\chi }_{4}^{2}=8.2$, *P* = 0.08), and (C) early-instar *Pn. pyralis* larvae (${\chi }_{1}^{2}=12.4$, *P* = 0.0004). Different letters indicate significant differences in feeding activity within each cohort at *P* < 0.05 (Benjamini–Hochberg correction for multiple comparisons).

Clothianidin exposure significantly reduced the number of times firefly larvae fed (Fig. 6). During the toxicity assays, no *Pn. pyralis* or *Pt. versicolor* larvae exposed to the highest clothianidin concentration (10,000 ng g^−1^ soil) fed. Late-instar *Pt. versicolor* exposed to 1,000 ng g^−1^ soil fed significantly less frequently than control larvae (*χ*^2^_4_ = 16.3, *P* = 0.003), and early-instar *Pn. pyralis* larvae fed significantly less at higher doses (1,000 ng g^−1^ and 10,000 ng g^−1^) compared to the control or lower doses (*χ*^2^_1_ = 12.4, *P* = 0.0004).

### Soil-chambers, molting, and pupation of late-instar *Photuris versicolor*

**Figure 7:**
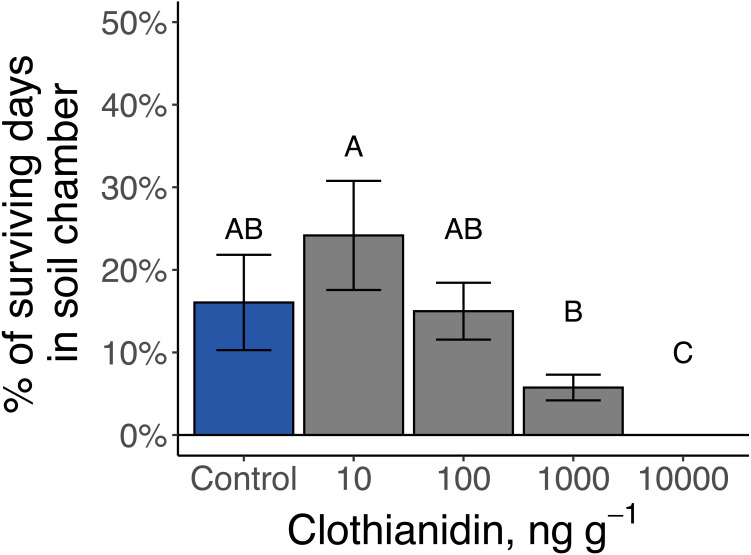
Amount of time that late-instar *Pt. versicolor* spent in soil chambers at different clothianidin-exposure levels (${\chi }_{4}^{2}=18.4$, *P* = 0.001). Different letters indicate significant differences at *P* < 0.05 (Benjamini–Hochberg correction for multiple comparisons).

The 14 late-instar *Photuris* larvae that survived as larvae through day 100 went through 1 to 5 periods of consecutive days when they regularly formed protective soil chambers (median = 2 periods) and spent anywhere from 1 to 20 total days in soil chambers (median = 9 d). Larvae exposed to 10,000 ng g^−1^ clothianidin never constructed soil chambers while larvae exposed to 1 ppm clothianidin spent significantly fewer days in soil chambers than larvae exposed to 10 ng g^−1^ (*P* = 0.01; Fig. 7).

Formation of protective soil chambers did not correspond with molting or pupation, and all recorded molting and pupation events occurred outside soil chambers, on the soil surface. Late-instar *Pt. versicolor* larvae only molted once or twice, irrespective of how frequently or for how long they built soil chambers (larvae that survived through 100 days; frequency: *R*^2^_*ad*__j_ = −0.09, *F*_1,10_ = 0.10, *P* = 0.76; duration: *R*^2^_*ad*__j_ = −0.02, *F*_1,10_ = 0.81, *P* = 0.39). Six of the thirty late-instar *Pt. versicolor* larvae pupated; five of which successfully eclosed within 35 d of starting the assay (two controls, one at 10 ng g^−1^, two at 100 ng g^−1^) and one which was unsuccessful (1,000 ng g^−1^). The unsuccessful larva failed to shed its last-instar exoskeleton and died during the pupal stage. At 35 d, three of the larvae exposed to the highest clothianidin concentration (10,000 ng g^−1^) were still alive, but none of these larvae ever entered a pupal stage. Of the individuals that successfully eclosed, three were lab-reared from eggs laid in 2019 (3 out of 5) while only two were wild-collected (2 out of 25).

## Discussion

*Photuris versicolor* complex and *Photinus pyralis* larvae did not significantly respond to clothianidin concentrations at or below 100 ng g^−1^ soil, but both firefly species exhibited significant toxic responses to higher concentrations. Although some of the larvae exposed to 10,000 ng clothianidin g^−1^ soil showed a toxic response within four hours of exposure, compared to other soil invertebrates, larvae of these two firefly species were relatively tolerant to clothianidin-treated soil. Twenty-four hour TC_50_ values for *Pt. versicolor* and *Pn. pyralis* were over 2 × and 30 × the TC_50_values for the earthworm *Eisenia andrei* and the collembolan *Folsomia candida*, respectively (de Limae Silva et al., 2020). Twenty-four hour LC_50_ values exceeded our maximum test concentration of 10^4^ ng clothianidin g^−1^ soil (10 ppm), indicating higher tolerance to clothianidin compared to other soil-dwelling beetles (*Agriotes* spp. [Elateridae] and *Atheta coriaria* [Staphylinidae]; (Van Herk et al., 2007; Cloyd et al., 2009)). The one other study which tested neonicotinoid toxicity to fireflies observed 13% survival of aquatic *Luciola lateralis* larvae after 24 h of exposure to 10^5^ ng thiamethoxam mL^−1^ in water (Lee et al., 2008); these results suggest that fireflies as a group may be somewhat tolerant to neonicotinoid exposure, although this is likely a tenuous conclusion because it is based on just two studies that represent less than 0.2% of all described firefly species (Lewis et al., 2020). Tolerance to neonicotinoids may partly explain why populations of *Pt. versicolor* and *Pn. pyralis* do not appear to be declining as fast as rarer firefly species (Reed et al., 2020), which may be more sensitive to neonicotinoid exposure. *Pt. versicolor* and *Pn. pyralis* may tolerate clothianidin exposure due to multiple behavioral, morphological, and biochemical processes that could limit their sensitivity to clothianidin (Alyokhin et al., 2008).

Behavioral avoidance of neonicotinoids has been observed across insect orders and beetle families (Easton & Goulson, 2013; Fernandes et al., 2016; Pisa et al., 2021; Korenko et al., 2019), and the results of this current study provide some support for behavioral avoidance of neonicotinoids by Lampyridae. Although firefly larvae could not avoid dermal exposure to the treated soil in our arenas, they may have decreased oral exposure by limiting construction of their soil chambers. To form soil chambers, *Pt. versicolor* larvae manipulate soil with their mouthparts (Buschman, 1984), providing a potentially more toxic pathway for neonicotinoid exposure (Decourtye & Devillers, 2010). Because neonicotinoids are repellant to other beetle species (Easton & Goulson, 2013), neonicotinoid-treated soil could have repulsed firefly larvae, possibly explaining reduced chamber formation above 1,000 ng clothianidin g^−1^ soil. Alternatively, sub-lethal neonicotinoid exposure may simply decrease the ability of fireflies to construct soil chambers. Choice-based avoidance studies could be used to test if avoidance or direct toxicity drove the decreased time late-instar *Pt. versicolor* spent constructing and inhabiting soil chambers at high-clothianidin concentrations.

In addition to behavioral avoidance, specific morphological and metabolic characteristics of fireflies may protect *Pt. versicolor* and *Pn. pyralis* larvae from toxic clothianidin exposure. Unlike many other soil invertebrates (e.g., earthworms and mollusks), firefly larvae have a comparably protective cuticle that may act as an effective barrier against neonicotinoid uptake (Decourtye & Devillers, 2010; Wang et al., 2012). And even when clothianidin is absorbed, insects can resist target-site exposure by quickly detoxify and/or excrete neonicotinoids (Olson, Dively & Nelson, 2000; Alyokhin et al., 2008). Although there has been no work on neonicotinoid metabolism by fireflies, *Pt. versicolor* and *Pn. pyralis* may upregulate detoxification enzymes after clothianidin exposure, similar to an aquatic firefly species after exposure to benzo[a]pyrene (Zhang et al., 2021). Additionally, *Pt. versicolor* and *Pn. pyralis* may be tolerant to clothianidin if neonicotinoids have a low binding affinity to target sites on firefly neurons. Neonicotinoids primarily target nicotinic acetylcholine receptors (nAChRs), which regulate cation movement and neuron firing in response to acetylcholine levels (Matsuda, Ihara & Sattelle, 2020). Neonicotinoid insecticides agonistically bind to these receptors, forcing ion channels open, leading to spasms and eventual paralysis (Simon-Delso et al., 2015). As neonicotinoids have broad activity across insect orders (Matsuda, Ihara & Sattelle, 2020), it is unlikely that clothianidin has a low binding affinity for nAChRs of *Pt. versicolor* and *Pn. pyralis*.

There is also the unlikely possibility that extensive neonicotinoid use has exerted selection pressure on the firefly populations in central Pennsylvania to evolve resistance to clothianidin. The way neonicotinoids are currently used is a perfect storm for developing insecticide resistance (Tooker, Douglas & Krupke, 2017), and while most concern has focused on resistance-development in herbivorous pest species, biocontrol agents and other predatory arthropods can develop insecticide tolerance and resistance in response to heavy insecticide use (Bielza, 2016; Mota-Sanchez & Wise, 2021). Although insecticide-resistance is thought to be rare among biocontrol agents, lady beetles (Coleoptera: Coccinellidae) in particular, have been found to develop resistance to a variety of broad-spectrum insecticides, including neonicotinoids (Tang et al., 2015). Insecticide resistance has not been studied in many non-pest species (including lampyrids), but if the selection pressure is high enough, firefly populations could evolve increased tolerance or even resistance to neonicotinoid insecticides.

Differences among any of these potential mechanisms are likely driving differences in tolerance between the two firefly species, namely, the dramatically reduced feeding response of *Pn. pyralis* to clothianidin exposure. Although this difference could have been exacerbated by mite pressure and the smaller body size of early-instar *Pn. pyralis*, it is possible that *Pn. pyralis* has higher uptake, higher active-site affinity, or lower metabolism of clothianidin as compared to *Pt. versicolor*.

Despite their relative tolerance to clothianidin exposure, field-realistic neonicotinoid concentrations may still pose a chronic threat to *Pt. versicolor* and *Pn. pyralis .* Although residual neonicotinoid concentrations in soil are often below 100 ng g^−1^ (Schaafsma et al., 2016; Radolinski et al., 2019; Pearsons et al., 2021), concentrations can regularly exceed these levels after agricultural applications (as high as 594 ng g^−1^ 23 days after planting neonicotinoid-coated seeds; (Radolinski et al., 2019)), after turf applications (3 × higher than in agronomic settings; (Armbrust & Peeler, 2002)) and after soil drenches to manage hemlock wooly adelgid (over 4,000 ng AI g^−1^ soil; Knoepp et al., 2012). Such high concentrations are well within the acutely toxic and chronically lethal range for *Pt. versicolor* and *Pn. pyralis* larvae (Table 2). Encountering such high concentrations are likely to be even more lethal under field conditions, as firefly larvae that exhibited toxic responses in the laboratory would be vulnerable to predation and starvation, two risks that can increase mortality from insecticides (Kunkel, Held & Potter, 2001). Additionally, further work is needed to assess if neonicotinoid exposure can exacerbate other stressors affecting firefly populations (i.e., light pollution) or if neonicotinoids pose a significant risk to firefly eggs or adults.

As observed with other predatory beetle species (*Cycloneda sanguinea* [Coccinellidae] and *Chauliognathus flavipes* [Cantharidae]; Fernandes et al., 2016, firefly larvae exhibited reduced feeding activity in response to high neonicotinoid exposure. Firefly larvae that feed less frequently may have less successful eclosion rates, and those that do eclose may have lower reproductive success. Additionally, the prey that fireflies encounter in neonicotinoid-contaminated environments likely provide an additional neonicotinoid exposure route. *Photinus* larvae primarily feed on earthworms (Lewis et al., 2020), which have been found to contain neonicotinoid concentrations above 200 ng g^−1^ when collected from soybean fields that were planted with neonicotinoid-coated seeds (Douglas, Rohr & Tooker, 2015) and 700 ng g^−1^ when collected from treated cereal fields (Pelosi et al., 2021). Firefly larvae of other species are known to feed on slugs (Barker, 2004), which can also contain high doses of neonicotinoids (500 ng g^−1^), leading to disrupted biological control provided by carabid beetles (Douglas, Rohr & Tooker, 2015). Compounded with reduced prey availability in habitats where neonicotinoids are used (Ritchie et al., 2019; Tooker & Pearsons, 2021), decreased feeding activity and high risks of further neonicotinoid exposure through contaminated prey may explain why adult lampyrid densities are significantly lower where clothianidin has been used as a seed coating (Disque et al., 2019), even if acute mortality is low. Adult fireflies may also encounter neonicotinoid residues while resting on sprayed vegetation or during oviposition into soil (Pisa et al., 2021), although the risk of such exposure does not appear to have been explored.

Despite low acute mortality, the sublethal effects of clothianidin were surprising, as some *Pt. versicolor* larvae survived in a severely intoxicated state (not feeding, not building protective soil chambers, only occasionally moving legs and/or mandibles) for over two months. A similar phenomenon has been observed in European wireworms (*Agriotes* spp. [Coleoptera: Elateridae]) after exposure to clothianidin, with individuals surviving and even recovering from a severely intoxicated state that can last months (Van Herk et al., 2007; Vernon et al., 2007). For pests like *Agriotes* spp., such sub-lethal effects of clothianidin exposure could still decrease crop damage but may exacerbate the risk of *Agriotes* spp. developing neonicotinoid resistance. For predators like *Pt. versicolor*, this long-term intoxication may limit their potential to provide biological control beyond what would be expected based on population declines.

## Conclusions

As larvae of the two firefly species that we studied appear to be somewhat tolerant to clothianidin-treated soil, neonicotinoids alone may not be significant direct factors in firefly declines in North America, at least for common species. Nevertheless, firefly populations around the world appear to be suffering from other stressors (e.g., habitat loss, reduced prey availability, light pollution), and ecological research has demonstrated that animal populations exposed to multiple stressors can suffer disproportionally more than what is suffered from a single stress (Relyea & Mills, 2001). Therefore, continued widespread contamination of larval firefly habitats with neonicotinoids may hold potential to exacerbate the influence of other stressors on declining firefly populations (Lewis et al., 2020). We encourage researchers with access to other species of fireflies, particularly those with declining populations in areas where neonicotinoids are commonly used, to explore their toxicological responses to insecticides.

## Supplemental Information

10.7717/peerj.12495/supp-1 Supplemental Information 1 Raw Data Firefly larvae acute responses, long-term survival, feeding behavior, soil chamber formation, and molting frequency under clothianidin exposure.

## Additional Information and Declarations

### Data Availability

The following information was supplied regarding data availability: The raw data is available in the Supplementary Files.
